# Effect of implant luting cements on the retention of cement-retained, implant-supported zirconia restorations fabricated using CAD-CAM technology – an *in vitro* study

**DOI:** 10.2340/biid.v12.45073

**Published:** 2025-12-17

**Authors:** Shreya Kabadi, Nayana Shriram Anasane

**Affiliations:** Department of Prosthodontics, Dr. D.Y. Patil Dental College and Hospital, Dr. D. Y. Patil Vidyapeeth (Deemed-to-be-University), Pune, India

**Keywords:** Cement retained prosthesis, fixed dental prosthesis, implant-supported restoration, luting cements, retention, Zirconia

## Abstract

**Objective:**

The study focused on drawing a comparative evaluation amongst four commercially available implant luting cements used to retain implant-supported zirconia restorations fabricated using a Computer-Aided Design-Computer-Aided Manufacturing (CAD-CAM) system.

**Materials and methods:**

A heat-activated polymethyl methacrylate (PMMA) model was fabricated to mount four implant abutment-analogue complexes. These complexes were scanned using a digital laboratory scanner to mill 60 zirconia copings using CAD-CAM software. The copings were divided into four groups depending on the type of implant luting cement used (*n* = 15). The zirconia copings were then cemented over titanium abutments and a tensile load at a crosshead speed of 1 mm/min was applied to the samples to perform a pull-out test using Universal Testing Machine. Thereafter, the load required to de-cement each coping was obtained, thus the retention for each coping was measured.

**Statistical analysis:**

One-way analysis of variance (ANOVA) was advocated to statistically analyse the results and the *post hoc* Bonferroni test was used for multiple comparison using Statistical Package for the Social Sciences (SPSS) software.

**Results:**

Multilink implant showed highest tensile strength (2.24 ± 1.01 kgf/mm^2^) being statistically significantly different from the other three cements (*p* < 0.001). This was followed by TgImplaCem (0.82 ± 0.32 kgf/mm^2^) and Temp-Bond NE (0.71 ± 0.13 kgf/mm^2^). The lowest tensile strength was exhibited by ImplaLute (0.31 ± 0.10 kgf/mm^2^). Intergroup comparison between TgImplaCem, Temp-Bond NE, and ImplaLute cements did not show statistically significant difference in tensile strength values (*p* < 0.001).

**Conclusion:**

It was concluded that both non-eugenol zinc oxide provisional luting cement (TgImplaCem) and dual-curing resin-based implant cement (Temp-Bond NE) can be advocated to cement implant-supported prostheses since they both permit adequate retention with ease of retrievability.

## Introduction

It is essential for the prosthodontist to recognise the importance of enhancing patient’s overall health to aid in maintaining the well-being of the oral structures. Implant-supported prostheses are predominantly fixed restorations, which can be advocated to replace either a single tooth or multiple teeth. These are divided into screw-retained prostheses and cement-retained prostheses [1–3]. The clinician decides the desired mode to retain the prostheses to the implant-abutment complex based on the clinical requirement, the outcome, and the aesthetics [3]. Although screw-retained prostheses are often the choice of retention in most cases, they have a few drawbacks such as active fit, prosthetic screw loosening, visible access holes, and the need for positioning of the implant to be optimal, which in effect compromises the aesthetics and occlusion [1]. Whereas, cement-retained prostheses show enhanced aesthetics, elimination of prosthetic screw loosening, and creation of a superior occlusion. However, their downsides include difficulty in retrieval of the prosthesis and cement excess [1, 2]. Owing to these factors, the choice of cement stands to be of utmost importance to aid in retention of the prosthesis, but also to promote feasibility of retrieval. Various implant-supported prostheses are prone to abutment screw loosening or fracture due to magnitude and direction of oral forces or parafunctional habits. It is very difficult to treat these screw related complications when cement-retained implant-supported prosthesis are cemented using permanent cement [4–6]. Hence, temporary luting cements are widely used for cement-retained prostheses [7, 8].

Various manufacturers have now introduced a variety of cements specially formulated for cementation of cement-retained implant-supported fixed dental prostheses [3]. Non-eugenol zinc oxide provisional luting cement, zinc oxide eugenol provisional cement, zinc oxide eugenol and non-eugenol acrylic, and resin-based temporary cements are the most preferred choice of cement for cementation of cement-retained implant-supported fixed dental prostheses [3, 7, 9–14]. Kapoor et al. studied the retention of implant-supported restorations cemented using five different luting agents and interpreted that non-eugenol acrylic based cement and temporary resin implant cement ensure ease of retrievability of the prosthesis [2]. There is limited data to compare the effect of implant luting cements on implant-supported computer-aided design and computer-aided manufacturing (CAD/CAM) restorations. The advantage of CAD/CAM systems is to enhance accuracy as they omit several fabrication steps followed during the casting procedure to fabricate conventional restorations. Another advantage is to get more uniform restorations with respect to thickness and cement space thus permitting standardisation [15, 16].

Considering the above-mentioned aspects, this *in vitro* study was conducted to evaluate the retention and ease of retrievability amongst four commercially available implant luting cements using zirconia copings fabricated using CAD/CAM technique cemented on implant abutments. The null hypothesis was that the implant luting cements would not show any significant difference in the retention of cement-retained implant-supported CAD/CAM restorations.

## Methodology

In the present study, a heat-activated clear polymethyl methacrylate (PMMA) model was fabricated to mount four implant abutment-analogue complexes. These complexes were scanned using three-dimensional digital laboratory scanner to mill 60 zirconia copings using CAD-CAM software. The copings were divided into four groups based on the cement types used, thus the sample size of each group was 15. The sample size was calculated based on previous literature with 95% confidence interval (CI) (*p* < 0.05 significance) and 80% power of the study with 0.2 error. Considering this, the sample size calculated was equal to 11.28 which was rounded off to 15 in each group.


$$n={\frac{{\text{Z}}_{1-\alpha }-2\beta \;6}{\delta }}^{2}$$

**Group I:** Zirconia copings cemented to implant abutments using non-eugenol zinc oxide provisional luting cement (Temp-Bond NE, Kerr Corporation, U.S.A.) (*n* = 15).

**Group II:** Zirconia copings cemented to implant abutments using dual-curing resin-based implant cement (TgImplaCem, Technical and General Ltd, 2 Albion Place, London, UK) (*n* = 15).

**Group III:** Zirconia copings cemented to implant abutments using resin-based acrylic urethane implant cement (ImplaLute, Medicept UK Ltd) (*n* = 15).

**Group IV:** Zirconia copings cemented to implant abutments using self-curing adhesive luting composite (Multilink Implant, Ivoclar Vivadent, Ontario, Canada) (*n* = 15).

The study involved the following steps:

- Fabrication of heat-activated clear PMMA model.
- Placement of implant analogue-abutment complexes in the model.
- Milling of zirconia copings using CAD/CAM software.
- Cementation of zirconia copings onto implant abutments using four different luting cements.
- Measuring tensile load to de-cement each coping using Universal Testing Machine.

### Fabrication of heat-activated clear PMMA model

A heat-activated clear PMMA model was fabricated using a standard edentulous mandibular mould (Nissin Dental Products Inc., Kyoto, Japan). The wax pattern was fabricated using hard wax (Carvex Set Up Hard, Haarlem, Netherlands). Once the wax pattern was obtained, heat-activated clear PMMA was used to process the model (Dental Product of India, Mumbai, India). Thereafter, the model was finished and polished using acrylic polishing burs (DFS, Germany), sand paper (John Oakey and Mohan, Uttar Pradesh, India), and polishing buff (Samit products, DentoKem, New Delhi).

### Placement of implant abutment-analogue complexes in the model

**Figure 1:**
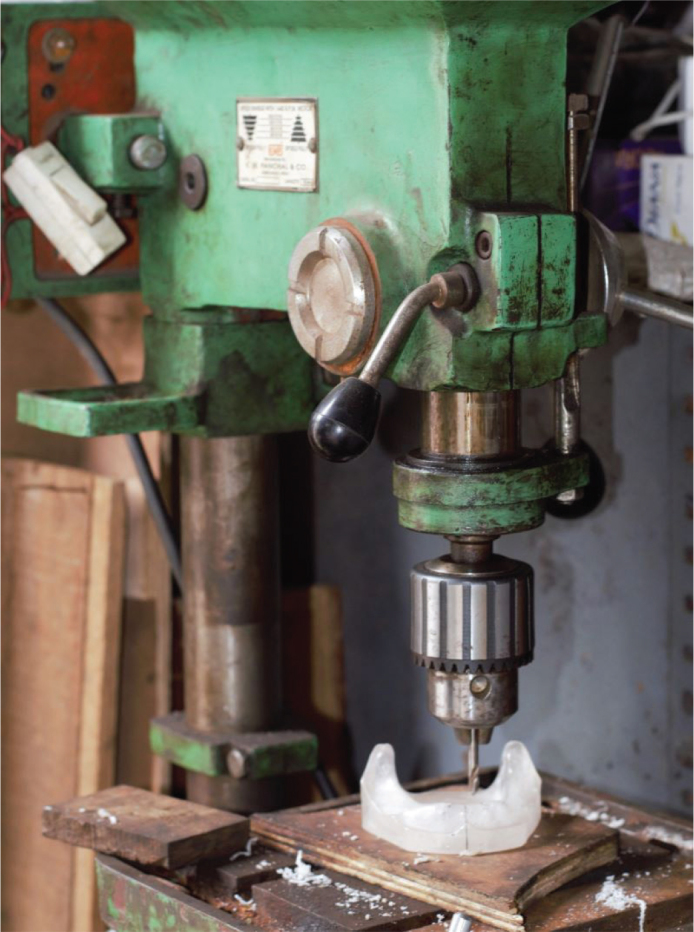
Pillar drilling machine.

The heat-activated clear PMMA model was stabilised on a flat platform and four recesses of 4.5 mm diameter × 12 mm length were drilled perpendicular to the plane of the model using pillar drilling machine (1,440 r.p.m motor pully, K.M. Panchal and Co., KMP, India) to house implant analogues (Genesis Implant, JJ Implants, Carlsbad, U.S.A.) (Figure 1). Two recesses were drilled in mandibular first molar region and two in mandibular canine region.

**Figure 2:**
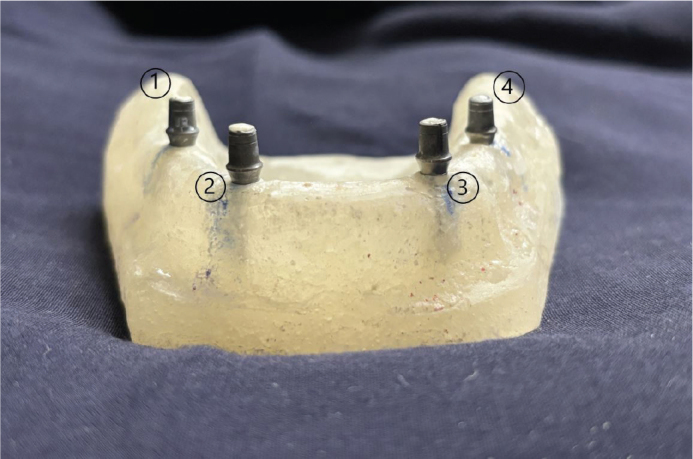
Implant analogue-abutment complexes marked as 1, 2, 3, and 4.

Four dental implant analogues of length 10 mm were aligned vertically into the analogue recesses prepared in the model so as to ensure parallelism of the complexes. The implant analogues were secured in each recess using clear auto-polymerising PMMA, thereby flushing the collar with the crest of the ridge. Thereafter, prefabricated straight titanium implant abutments of 4.5 mm diameter, 5.5 mm stump height with 1.5 mm of gingival height were fastened to the implant analogues using hex driver (Genesis Implant, JJ Implants, Carlsbad, U.S.A.) and torque wrench (Genesis Implant, JJ Implants, Carlsbad, U.S.A.) at 30 N-cm of torque. Implant analogue-abutment complexes marked as 1, 2, 3, and 4, respectively were used for four different groups of cements as per the groups mentioned above (Figure 2).

### Milling of zirconia copings using CAD/CAM software

**Figure 3:**
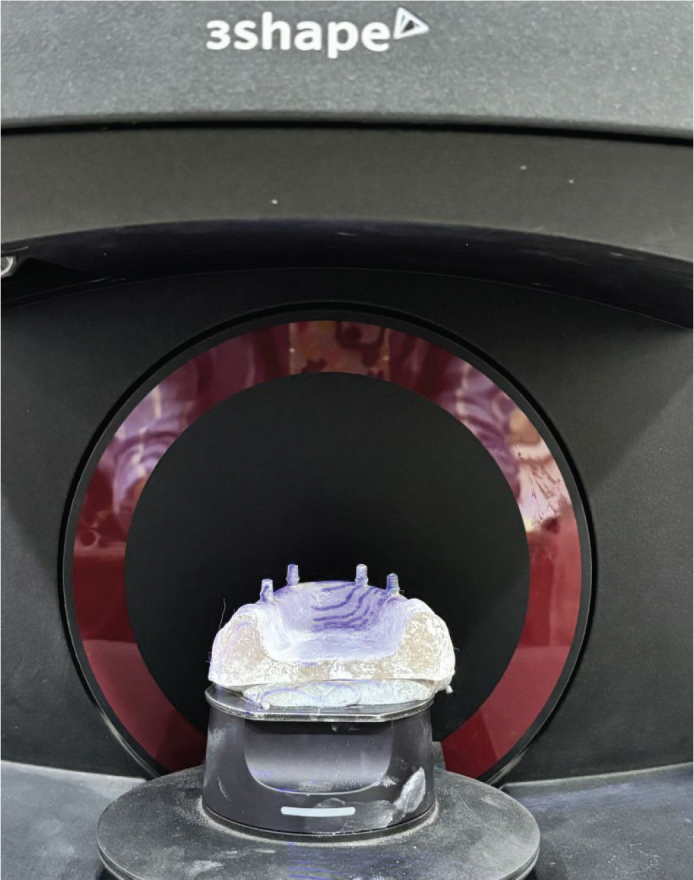
Scanning of implant abutments using digital lab scanner.

Implant abutments were scanned using three-dimensional digital lab scanner (3Shape, E1 TRIOS 4, Copenhagen C, Denmark) (Figure 3). Sixty zirconia copings were fabricated using CAD-CAM system. Dental Zirconia blank of ST series with 12 mm thickness was used to fabricate the copings (Shenzhen Upcera Dental Technology Co., Ltd, Guangdong, China). These copings were fabricated with a gap of 0.02 mm for the cement space and with the coping thickness of 0.8 mm. A loop attachment of 2 mm thickness and 2 cm diameter was incorporated on the occlusal surface of each coping. All the zirconia copings were further treated for airborne particle abrasion which involved propelling 50 μm aluminium oxide particles against the zirconia copings under 0.2 MPa pressure. The same implant-abutment complex with 15 different zirconia copings were used for each group of luting cements.

### Cementation of zirconia copings onto implant abutments using four different luting cements

The fabricated zirconia copings were divided into four equal groups depending on the type of cements used for cementation of copings with each group comprising 15 copings (*n* = 15). Prior to cementation, the abutment screw access hole was covered using putty impression material to prevent seepage of cement into the same. Thereafter, each coping was bonded to the implant abutment using the respective cements as per the manufacturer’s instructions. The same implant-abutment complex with 15 different zirconia copings were used for each group of luting cement. Copings in Group I were cemented using non-eugenol zinc oxide provisional luting cement. Copings in Group II were cemented using dual-curing resin-based implant cement. Copings in Group III were cemented using resin-based acrylic urethane implant cement, whereas, copings in Group IV were cemented using self-curing adhesive luting composite.

Thereafter, a 10 second-long firm finger pressure was applied to seat the copings on the respective abutments, followed by application of constant axial compressive load of 2 kg for 3 min. Excess cement was removed by means of an explorer, after which the samples were stored in artificial saliva (Nanochemazone Inc, Kaithal, Haryana) for 7 days at 37°C to simulate oral environment and the impact it will have on the characteristics of luting cements were tested in the present study.

### Measuring tensile load to de-cement each coping using universal testing machine and statistical analysis

**Figure 4:**
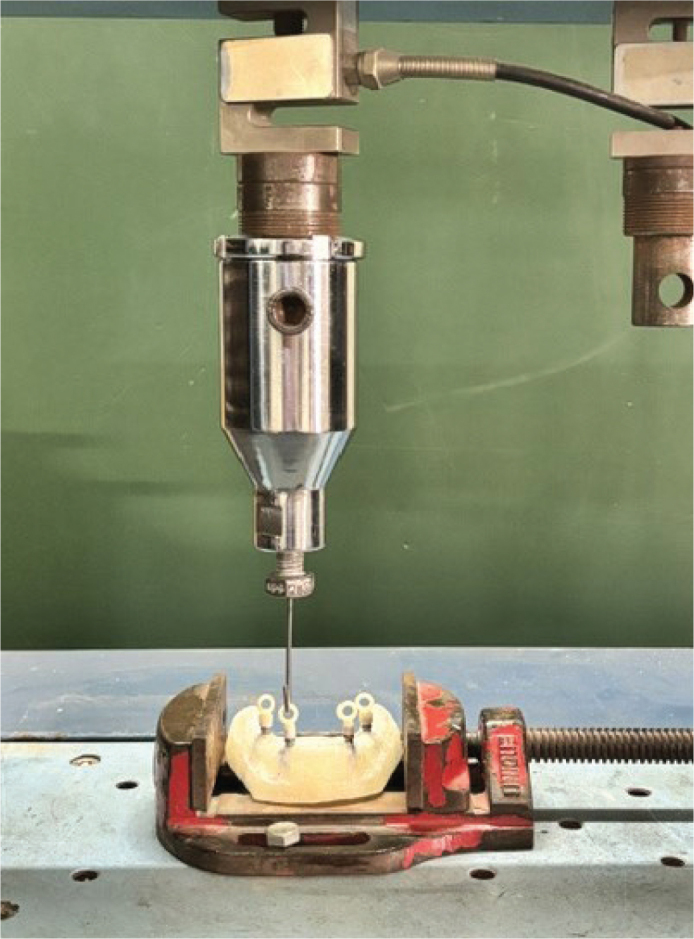
Tensile loading test using Universal Testing Machine.

After the ageing process, the Universal Testing Machine (UNITEST 10, ACME Engineers, Pune, India ) was used to measure the dislodging forces of each coping. The coping was secured to the holding device of the Universal Testing Machine to perform tensile loading test under static conditions (Figure 4).

Tensile load at a crosshead speed of 1mm/min was applied to the samples. The measurement of load required to de-cement each coping was obtained in Newton (N). This load was divided by the surface area of the abutment (4.5 mm × 5.5 mm) to get the tensile strength in the unit of kgf/mm^2^. Tensile load testing was carried out to de-cement 15 zirconia copings for each group of luting cements. The same implant-abutment complex with 15 different zirconia copings were used for each group of luting cements. As same implant-abutment complexes were used for 15 zirconia copings within each group of cement, once the first zirconia coping was de-cemented, residual cement from each abutment was removed using an explorer, and the abutments were placed in an ultrasonic cleaner for 15 min. The abutments were thereafter thoroughly cleaned in distilled water and air dried to permit complete removal of residual cement. After ensuring total removal of cements by visual inspection, the abutments were placed into the analogues again and the next set of copings were cemented and tested similarly, and the results were tabulated.

### Statistical analysis

Statistical Package for the Social Sciences (SPSS) software version 19-SPSS Inc (Chicago, IL, USA) was used to process and analyse the data. The mean and standard deviation of each group was determined and one-way analysis of variance (ANOVA) was advocated to statistically analyse the result and Bonferroni *post hoc* test was used for multiple comparison. While we considered 95% CI in our analysis, *p* < 0.05 was considered statistically significant. Single blinding was done during assessment of the result. The primary outcome of the study was retention of the prosthesis, while the secondary outcome of the study was retrievability of the prosthesis.

## Results

**Figure 5:**
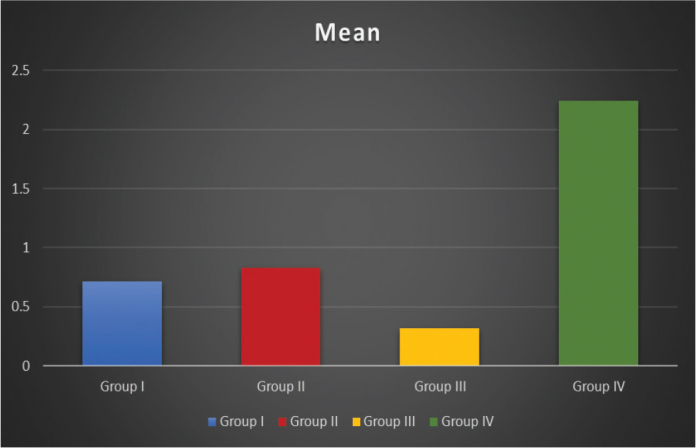
Graphical representation of tensile strength values.

The results of the present study found highest mean score for Group IV (2.24 ± 1.0 kgf/mm^2^), followed by Group II (0.82 ± 0.32 kgf/mm^2^), Group I (0.71 ± 0.13 kgf/mm^2^), and Group III (0.31 ± 0.10 kgf/mm^2^) (Table 1) (Figure 5). Thus, it was observed that Group IV cement (Multilink Implant) required the highest tensile strength to de-cement the copings, followed by Group II (TgImplaCem) and Group I (Temp-Bond NE). The least tensile strength to de-cement the copings was exhibited by Group III cement (ImplaLute).

**Table 1:** Values of tensile strength (kgf/mm^2^) required to de-cement copings from each group of luting cement.

	Group I (TempBond NE) (*n* = 15)	Group II (TgImplaCem) (*n* = 15)	Group III (ImplaLute) (*n* = 15)	Group IV (Multilink Implant) (*n* = 15)
**Mean values**	0.7137	0.8297	0.316	2.243
**Std. deviation**	0.1391584	0.3254960	0.1066242	1.0194092

Comparison of mean retentive strength amongst four different groups using one-way ANOVA showed a statistically significant difference (*F* = 14.430, *p* < 0.001) (Table 2).

**Table 2:** Distribution and comparison of mean retentive strength amongst four different groups using one-way ANOVA.

Groups	Mean	Std. deviation	95% confidence interval for mean		*F*	*P*
			Lower bound	Upper bound		
I	0.713783	0.1391584	0.567746	0.859821	14.430	< 0.001
II	0.829767	0.3254960	0.488180	1.171354		
III	0.316333	0.1066242	0.204438	0.428229		
IV	2.243117	1.0194092	1.173312	3.312921		
Total	1.025750	0.8994032	0.645965	1.405535		

*P* < 0.001.

Intergroup comparison using Bonferroni *post hoc* test showed a statistically significant difference between Groups I and IV with a mean difference 1.52 (*p* = 0.001), Groups II and IV with a mean difference 1.41 (*p* = 0.001), and Groups III and IV with a mean difference 1.92 (*p* < 0.001). Thus, Group IV cement showed statistically significant difference with all other three groups of cements.

Whereas, intergroup comparison amongst all other three groups that is Group I, II, and III showed no statistically significant difference although the difference was clinically significant (Table 3).

**Table 3:** Intergroup comparison using Bonferroni *post hoc* test.

(I) Group	(J) Group	Mean difference (I-J)	Std. error	*P*	95% confidence interval	
					Lower bound	Upper bound
I	II	−0.1159833	0.3130331	1.000	−1.032268	0.800302
	III	0.3974500	0.3130331	1.000	−0.518835	1.313735
	IV	−1.5293333*	0.3130331	0.001	−2.445618	−0.613048
II	III	0.5134333	0.3130331	0.700	−0.402852	1.429718
	IV	−1.4133500*	0.3130331	0.001	−2.329635	−0.497065
III	IV	−1.9267833*	0.3130331	< 0.001	−2.843068	−1.010498

* The mean difference is significant at 0.05 level.

## Discussion

Implant-supported prostheses are categorised into screw-retained and cement-retained types, with the latter being commonly employed in dentistry due to its similarity to traditional prosthodontic procedures. Cement-retained prostheses offer several advantages such as superior occlusion, reduced cost, aesthetic properties, passive fit, simplified component complexity and laboratory procedures, and decreased chair-side time [1–3, 17]. The selection of cement is crucial for achieving good retention and strength, while ensuring ease of retrieval and maintaining the sustainability of the implant prostheses. Provisional luting cements are usually advocated for this purpose, with their retention influenced by factors similar to those affecting natural teeth [1, 3, 18]. Nevertheless, the favoured method of securing the prosthesis is typically a deliberate decision made by the healthcare provider, considering the specific requirements of the clinical scenario or the intended result.

The type of luting agent used, amount of cement space fabricated in the prosthesis, and masticatory forces subjected are a few considerations affecting the overall health and retention of the implant restorations [2, 3, 19–22]. Although one might expect permanent luting cements to offer the highest retention, their effectiveness differs when used on implants compared to that when used on natural teeth. Various factors such as abutment surface preparation and treatment, height, and taper also impact the retention of these prostheses [7–9, 23–26]. An ideal cement should possess certain qualities such as optimum retention of the restoration, yet permit easy retrievability whenever desired.

Retrievability is the prime requisite for implant luting cements used for cement retained implant-supported prosthesis. In two piece implant system, abutment screws are prone to screw loosening or fracture due to magnitude and direction of oral forces, occlusion, parafunctional habits, and limitations of the components. Screw related complications are very difficult to treat when permanent cement is being used for cementation of cement retained implant-supported prosthesis. On the other hand, the implant luting cements should also possess optimum retentive values to provide adequate retention and to prevent embarrassment for the patient [4–6]. Consequently, manufacturers have introduced specially formulated implant luting cements to meet these criteria [3].

Therefore, this study was designed to evaluate the tensile strength of four newer commercially available implant luting cements such as non-eugenol zinc oxide provisional luting cement (TempBond NE), dual-curing resin-based implant cement (TgImplaCem), resin-based acrylic urethane implant cement (ImplaLute), and self-curing adhesive luting composite (Multilink Implant). In the present study, 60 zirconia copings were fabricated using CAD-CAM technology and were divided into four groups based on the type of cement used. To ensure standardisation, CAD-CAM techniques were used for fabricating specimens with a standard dimension and cement space.

In this study, all the copings were fabricated with 20 μm cement space for all the cements tested. Mehl et al. stated that with increase in cement thickness from 15 μm to 50 μm, crown retention decreases [27]. Abu-Obaid also studied the effect of cementation procedure and cement film thickness on the retention of implant-supported prosthesis and concluded that a cement thickness of 20 μm is optimal for increasing the retention of cemented restorations. This could be due to the lack of micromechanical interlocking and uneven cement distribution in larger space [28].

The fabricated zirconia copings were luted using four different cement groups. The tensile load required to de-cement these copings were measured using universal testing machine. Tensile strength recorded for non-eugenol zinc oxide provisional luting cement (Temp-Bond) and dual-curing resin-based implant cement (TgImplaCem), showed moderate retentive values. Amongst these two cements evaluated, dual-curing resin-based implant cement is more retentive than non-eugenol zinc oxide provisional luting cement, but the difference is not statistically significant. Hence, both the group of cements can be advocated for cementation of implant-supported prostheses since it permits ease of retrievability while also delivering good retention to the same. Similar results were obtained by Kapoor et al. for non-eugenol acrylic based temporary implant cement and non-eugenol temporary resin implant cement who also concluded that these cements would permit easy retrievability of the prosthesis [2]. Hendi et al. in 2023 also compared the marginal leakage and retention of milled zirconia and cobalt chromium copings cemented over implant abutments using different luting cements. The retentive strength values calculated for Temp-Bond cement was least amongst five different cements tested which contradicts with the result obtained in the present study. This variability in the result could be due to the use of diverse cement brands in both the studies [29].

The study showed highest tensile strength for self-curing adhesive luting composite (Multilink Implant), low solubility in water and high mechanical strength enable this material to establish a durable bond between the restoration and the abutment. Thus, making it the most retentive which is not advisable as an ideal implant luting cement. According to Worni et al., if the luting cement has high adhesive strength, superstructure can be damaged during removal of the prosthesis; hence, implant luting cements should permit ease of retrievability when desired [30]. This finding is similar to the results obtained by Volkmann et al. who evaluated the retentive force of zirconia crowns cemented over implant abutments having two different abutment heights using four different luting cements. The self-adhesive composite cement depicted highest pull-off force amongst four luting cements tested [31].

The study also highlighted the least retentive strength of resin-based acrylic urethane implant cement (ImplaLute) when compared to the rest of the cements. The increased film thickness of resin-based acrylic urethane cement may have compromised its physical properties, leading to reduced retention and marginal leakage [27, 28]. Similar result was obtained by Sarfaraz et al. [3] who evaluated the three different implant luting cements and found least retention with resin-based acrylic urethane cement. Thus the null hypothesis was not supported since all the four cements tested showed varied retentive values.

Worni in 2015 studied the retrievability of crowns cemented over implant abutments using three different luting agents and concluded that cemented implant crowns could be retrieved with different types of cements but the strength of definitive cement appears to be high [30]. Clinicians should carefully consider cement choice, particularly in cases with a high risk of component loosening, where weaker cements may be ineffective. The clinician should study each case thoroughly to make the correct choice for selection of cements for implant restorations and bear in mind that the selected cement should provide good retention but also allow ease of retrieval whenever necessary [32–33].

The present study was an *in vitro* study and the results obtained may not correlate clinically as desired. The reuse of the same implant abutment-analogue complex for multiple copings within each group could be a confounding variable due to material fatigue or incomplete cement removal. This could be the limitation of this study. Further research is required to explore more luting agents for different implant types simulating clinical conditions.

## Conclusion

Within the limitations of this *in vitro* study, the following conclusions were inferred:

- Tensile strength recorded for non-eugenol zinc oxide provisional luting cement and dual-curing resin-based implant cement showed moderate retentive values. Hence, it is correct to conclude that both the cements can be used to cement implant-supported restorations since it permits ease of retrievability with adequate retention.
- Amongst the four cements tested, self-curing adhesive luting composite showed the highest retentive values and thus the least retrievability.
- Resin-based acrylic urethane implant cement was the least retentive cement which could result in frequent dislodgement of the prosthesis.
